# 5-HT attenuates chronic stress-induced cognitive impairment in mice through intestinal flora disruption

**DOI:** 10.1186/s12974-023-02693-1

**Published:** 2023-02-03

**Authors:** Junxing Ma, Ran Wang, Yaoxing Chen, Zixu Wang, Yulan Dong

**Affiliations:** 1 National Key Laboratory of Veterinary Public Health Security, College of Veterinary Medicine, China Agricultural University, Beijing, 100193 China; 2 Key Laboratory of Precision Nutrition and Food Quality, Ministry of Education, Department of Nutrition and Health, China Agricultural University, Beijing, 100193 China

**Keywords:** CUMS, Hippocampus, 5-HT, Cognitive impairment, Gut microbiome

## Abstract

**Background:**

The microbiota–gut–brain axis plays an important role in the development of depression. The aim of this study was to investigate the effects of 5-HT on cognitive function, learning and memory induced by chronic unforeseeable mild stress stimulation (CUMS) in female mice. CUMS mice and TPH2 KO mice were used in the study. *Lactococcus lactis* E001-B-8 fungus powder was orally administered to mice with CUMS.

**Methods:**

We used the open field test, Morris water maze, tail suspension test and sucrose preference test to examine learning-related behaviours. In addition, AB-PAS staining, immunofluorescence, ELISA, qPCR, Western blotting and microbial sequencing were employed to address our hypotheses.

**Results:**

The effect of CUMS was more obvious in female mice than in male mice. Compared with female CUMS mice, extracellular serotonin levels in TPH2 KO CUMS mice were significantly reduced, and cognitive dysfunction was aggravated. Increased hippocampal autophagy levels, decreased neurotransmitter levels, reduced oxidative stress damage, increased neuroinflammatory responses and disrupted gut flora were observed. Moreover, *L. lactis* E001-B-8 significantly improved the cognitive behaviour of mice.

**Conclusions:**

These results strongly suggest that *L. lactis* E001-B-8 but not FLX can alleviate rodent depressive and anxiety-like behaviours in response to CUMS, which is associated with the improvement of 5-HT metabolism and modulation of the gut microbiome composition.

**Supplementary Information:**

The online version contains supplementary material available at 10.1186/s12974-023-02693-1.

## Introduction

In the past few years, accumulating evidence has indicated a relationship between gut microbes and hosts, including the gut–brain axis [1]. The gut–brain axis, a bidirectional communication system between the gastrointestinal tract and the central nervous system, is involved in the maintenance of homeostasis [2]. Gut microbes play a vital role in human health, and they affect the blood‒brain barrier (BBB), myelin sheath, neurogenesis, and other neurodevelopmental processes, many of which are mediated by metabolites produced by gut microbes [3]. Lactic acid bacteria (LAB), such as bacteria belonging to the *Lactobacillus* and *Bifidobacterium* genera*,* are considered probiotics that exert beneficial effects on gut homeostasis. In addition, a subgroup of probiotics also shapes CNS function and host behaviours through gut–brain axis (GBA) and has, therefore, been termed psychobiotics [4]. Evidence is emerging that certain probiotic strains of *Lactobacillus*, such as *L. rhamnosus* [5], *L. reuteri* [6], *L. plantarum*, and *L. paracasei*, and *Bifidobacterium*, including *B. longum* and *B. breve*, exert beneficial roles in alleviating symptoms of depression and restoring hippocampal 5-HT levels [7–9]. Certain probiotics, such as *Bifidobacterium longum subsp*. *infantis* E41 and *Bifidobacterium breve* CCFM1025 [10, 11], have been reported to alleviate depressive and anxiety-like symptoms in rodents by modulating the peripheral 5-HTP level, depicting a humoral route of the GBA through which a probiotic strain can affect central serotonergic function and host behaviour. *Lactococcus* is one of the genera within LAB, and the species *Lactococcus lactis* has been used for centuries in the fermentation of food, such as yogurt, cheese, and sauerkraut [12]. Certain *L. lactis* strains, such as *L. lactis subsp*. *cremoris* FC and *L. lactis subsp. lactis* NCDO 2118, have beneficial effects on gut health and have been demonstrated to ameliorate colitis in mice through immunomodulatory activity [13, 14]. A recent study highlighted that *L. lactis *subsp.* cremoris* LL95 can improve depressive/anxiety-like behaviour in female mice [15], indicating the possible antidepressant- and anxiolytic-like activities of certain *L. lactis* strains.

Major depressive disorder (MDD) involves neuropsychological alterations not only in emotional responses but also across a broad dimension of cognitive function. Symptoms of MDD include memory deficits, attention impairment [16–18], alterations in the speed of mental processing and motor performance and executive dysfunction [19–21]. Evidence suggests that 5-HT plays an important role in the control of anxiety and fear responses [22–27]. Serotonin is a major neurotransmitter in the central nervous system (CNS), and its role in psychiatric disorders is well-documented. TPH2 catalyses the rate-limiting step in the synthetic pathway for brain serotonin and is considered key for maintaining normal serotonin transmission in the CNS. Mice with targeted inactivation of TPH2 have provided insights into the role of 5-HT in the modulation of anxiety-like behaviours [28]. Previous studies of lifelong deficiency of brain 5-HT synthesis are consistent with the hypothesis that the brain serotonergic system plays an important role in the control of anxiety-like behaviours, fear learning, and behavioural responses to stress [29], effects that might be due to alterations in GABAergic transmission [30, 31]. Furthermore, mice with defects in 5-HT system development leading to a reduction in 5-HT neurons showed differential anxiety-like behaviours and fear memory [32–37]. Indeed, the 5-HT system is thought to play an essential role in the regulation of fear memory in rodents [27, 38]. Studies in animals demonstrate a direct anatomical connection between the main sources of serotonin in the brain, the brainstem dorsal and median raphe nuclei and forebrain limbic structures, such as the medial prefrontal cortex, hippocampus, and amygdala, that control anxiety and fear responses [25, 39–41]. Of particular interest to contextual fear conditioning is the dorsal hippocampus [42], which receives serotonergic projections primarily from the median raphe nucleus [43–45]. The hippocampus is a critical brain structure responsible for cognition, learning and memory and is particularly vulnerable to uncontrollable stress [46–48]. The hippocampus is composed of three subregions: the dentate gyrus (DG), CA1, and CA3 [49]. Repeated exposure to stress produces a negative effect on the structure and function of the hippocampus [50, 51]. Multiple studies have shown that damage to the hippocampus causes defects in spatial learning or episodic memory [52, 53]. However, whether 5-HT in the hippocampus is involved in the social cognitive impairment induced by CUMS remains unclear.

Although 5-HT influences gut functions, including motility function, it is unable to cross the mature blood–brain barrier and interact with neural tissue. In this study, *L. lactis* E001-B-8 (which promotes 5-HT concentration) may alleviate rodent depressive and anxiety-like behaviours on chronic stress-induced cognitive impairment in mice, and its mechanisms of action were demonstrated by analysing the correlations between the above indicators and the cognitive memory and depression behaviour of mice.

## Materials and methods

### Animals and drug treatment

Adult female C57BL/6J mice (8 weeks of age; Vital River Laboratory Animal Technology Co., Ltd., Beijing, China) were used in this study. TPH2 KO mice were donated by Beijing Center for Brain Science and Brain-like Research. TPH2 is the initial and rate-limiting enzyme of 5-HT in the central nervous system. All mice were analysed on the C57BL6/J background. All animals were kept at a temperature of 22 ± 1 °C with 12 h light/12 h dark cycles, and food and water were provided ad libitum. After adaptation for 2 weeks, the animals were housed under a 12 h light/dark cycle (lights on at 8:00 a.m.), and food and water were available ad libitum. The mice were fed, watered and weighed daily. Behavioural tests were conducted as scheduled after 3 weeks of feeding, and mice were sacrificed after the last behavioural test and blood glucose measurement. All animal procedures were approved by the Animal Care and Use Committee of China Agricultural University (AW03502202-2-1), and all efforts were made to minimize the number of animals used and their suffering.

The mice were first divided into female mice and male mice were selected, and after 7 days of adaptive feeding, to explore the effect of CUMS on male and female mice, the following experiments with wild-type male and female mice groups, they were randomly divided into control group, CUMS group and CUMS + FLX group (*n* = 10 each group). The CUMS lasted for 21 days, and on the 22nd day behavioral tests were performed. After the behavioral experiment all mice were euthanized under anaesthesia using 10% chloral hydrate. Their serum, hippocampus, colon tissue and colonic content were harvested. To further explore the effect of 5-HT on CUMS mice, to design (1) WT control group, (2) WT CUMS group, (3) WT CUMS + FLX (20 mg/kg) group, (4) TPH2 KO group, (5) TPH2 CUMS group. Finally, to explore the effect of *Lactococcus lactis* E001-B-8 (promoting 5-HT synthesis in intestine) on CUMS mice, the mice were divided into (1) Control group, (2) CUMS + Vehicle group, (3) CUMS + FLX group, (4) CUMS + *Lactococcus lactis* E001-B-8 group. The animals were socially housed and left undisturbed unless necessary procedures including routine cleaning.

### Behavioural tests

#### Sucrose preference test

The sucrose preference test (SPT) was performed at the same time before, 1 week after, 2 weeks after and 3 weeks after the start of the modelling stimulus, and SPT data were collected. The mice were acclimatized to sugar-containing water for 1 h. Two water bottles, one bottle of 1% sucrose water solution and one bottle of pure water, were placed in each cage for mice to drink. To avoid mice preferentially drinking from the water bottle on one side, the water bottles were inverted after half an hour, and the consumption of sugar water and pure water was recorded. The mice were fed freely before the experiment, and tap water and sugar water consumption were measured by measuring the weight of the bottle. Sugar water preference was calculated based on the percentage of sugar water consumed in the overall fluid consumption: sugar water preference value = sugar water consumption (g)/[sugar water consumption (g) + water consumption (g)] × 100%.

#### Forced swimming test

Mice were placed into a clear Plexiglas cylinder (25 cm in height and 10 cm in diameter) filled up to two-thirds with water (24 °C) for a 5-min session. The sessions were video-recorded, and the duration of immobility was measured upon viewing the video recordings. Immobility was defined as the lack of active movements except for those required for floating.

#### Tail suspension test

The modified method was used for the tail suspension test (TST). The mice were hung individually with paper adhesive tape on a bar 35 cm above the table. The adhesive tape was placed 2–3 cm above the tip of the tail, and the mice were hung for 5 min. The duration of immobility was measured and recorded by trained observers. The mice were considered immobile when they showed no body movement during the test. Immobility reduction was regarded as an antidepressant activity.

#### Open field test

The OFT was performed in a box (50 cm × 50 cm × 50 cm). Each mouse was placed in a corner at the start of the test and recorded for 5 min by a camera located above the box. We cleaned the device with 75% alcohol after each trial. Sassafras was dried with paper towels until the air was dry, then the mice were replaced and the experiment was repeated [54]. A tracking system with an automated analysis system recorded the number of entries into the center zone, the time spent in the center zone and the total distance traveled (SMART 3.0, Panlab S.L.U., Spain). The center area of the open field apparatus was 25% of the total area (a square of approximately 25 cm × 25 cm).

#### Morris water maze

The test was conducted using a Morris water maze [55]. Mice were single-caged and brought into the testing room prior to the beginning of the experiment. The MWM test was conducted in a circular tank (diameter, 140 cm; height, 50 cm) (Shanghai Mobildatum Technology Co., Ltd.) in a dimly lit room. The water temperature was kept at 22–25 °C to inhibit the mice from floating. A submerged escape platform (10 × 10 cm) was equipped 1.5 cm below the milky water surface in one of the quadrants. Spatial cues of different geometry were decorated by the pool sides to help the mice recognize the platform position. The mice were individually handled for 1 day before starting the acquisition training. The mice were trained over 5 consecutive days with four trials per day per mouse. The trial was completed as soon as the mouse found the platform or when 60 s had elapsed. If the mouse cannot find the submerged platform on a given trial, the mouse was guided to the submerged platform. The latency and path to the platform were tracked and recorded. The swim speed was measured to analyze the involvement of motor function as a confounding factor. On day 6, a single probe test was performed to measure the integrity and strength of spatial memory 24 h after the last trial of the acquisition phase.

### Analysis of differential gene expression in the hippocampus of depressed mice by bioinformatics

On the National Center for Biotechnology Information (NCBI) website, the Gene Expression Omnibus was searched with the keyword “Major Depressive Disorder”. GSE151807 (https://www.ncbi.nlm.nih.gov/gds/?term=Major+Depressive+Disorder) was obtained by setting the mouse tissue and gene expression matrix as filtering criteria. The data set was based on high-throughput sequencing to detect gene expression levels in hippocampal tissues of mice with chronic mild stress-induced depression and control mice. This study was based on the screening of differentially expressed genes in the depression and control groups. We screened for differential gene expression based on fold change and *P* values in the hippocampal tissue of depressed and control mice. The differentially expressed genes (DEGs) were analysed using R software and the Limma function package in Bioconductor, and the screening conditions were set as follows: |log2FC|> . Gene set enrichment analysis was performed on the gene expression matrix of GSE151807. Gene Ontology (GO) enrichment analysis was performed using the online analysis tool DAVID for differential genes in the hippocampus of depressed mice. The data were visualized using R software and the ggplot2 functional package. The analysis was performed in terms of biological process (BP), cellular component (CC), and molecular function (MF). KEGG signalling pathway enrichment analysis was performed using DAVID for differentially expressed genes in the hippocampal tissue of depressed mice.

### AB-PAS staining

The colonic segments were immediately fixed in 4% paraformaldehyde in 0.1 M phosphate-buffered saline (pH 7.4, 4 °C) for 48 h and embedded in paraffin for sectioning (5 μm cross section). The tissue sections were stained with periodic acid-Schiff (AB-PAS). In the colon, at least 30 random fields in six sections of each sample with AB-PAS staining were photographed at × 400 magnification with a microscope (BX51; Olympus, Tokyo, Japan). The number of goblet cells per μm^2^ was calculated.

### Enzyme-linked immunosorbent assay (ELISA)

Hippocampal samples were collected for the detection of 5-HT concentrations using a competitive enzyme-linked immunosorbent assay (Uscn Life Science, Inc., Wuhan, China). All tests were performed according to the manufacturer’s instructions. Eight hippocampal samples were measured in each group. Each sample was tested in triplicate.

### Immunofluorescence

Staining was performed on 5 µm paraffin-embedded sections of mouse colon or brain tissue. After dehydration, slides were incubated in Antigen Unmasking Solution Citrate Buffer pH 6 (Vector Labs) for 20 min at 100 °C in a steamer for antigen retrieval and then blocked for 1 h at room temperature in 10% donkey serum. Staining was performed with primary antibodies overnight at 4 °C. Primary antibodies (PSD95, 1:500; BDNF, 1:500; DCX 1:500; NeuN 1:500; Claudin-1, 1:500. Abcam, Cambridge, MA, USA) were recognized by secondary antibodies diluted at 1:1000 in PBST and incubated for 1 h at room temperature. Nuclei were stained with DAPI (Abcam, Cambridge, MA, USA) for 10 min at room temperature, and all slides were cover-slipped with mounting media (Life Technologies) and imaged using a Nikon Eclipse 90i epifluorescence microscope (Nikon, Tokyo, Japan).

### RNA extraction and real-time quantitative PCR

Total RNA was extracted using TRIzol reagent (CW0580A, CoWin Biotech Co., Inc., Beijing, China). The concentration and purity of the extracted RNA were measured with a Nano Photometer (P330, Implen, Munich, Germany). The concentration of RNA was 1500–1800 ng/μL. Afterward, 2 μg of total RNA was mixed with reverse transcriptase, and other reagents from cDNA were synthesized using the GoScriptTM Reverse Transcription System (A5001, Promega, Madison, WI., USA). cDNA was diluted 10 times to conduct polymerase chain reactions. The primers used were synthesized by Invitrogen Trading of Shanghai, and qPCR amplification was performed with a Roche LightCycler 480 (Roche, Basel, Switzerland). Primers of qPCR efficiency with one specific melting peak were used for the analyses (the sequence of primers can be seen in Table 1). In this experiment, we used a 20 μL system containing 0.4 μL of forward primers, 0.4 μL of reverse primers, a 10 μL SYBR Green qPCR Master Mix kit (without ROX) (Q121-02, Vazyme Biotech Co., Ltd., Nanjing, China), 7.2 μL of nuclease-free water and 2 μL of cDNA. Relative target gene expression was obtained by normalizing the result to β-actin. Four replicates were tested for each sample to ensure the accuracy of the relative expression of the target gene in the sample. After amplification, according to the system-generated Ct value, the 2^−△△Ct^ method was used with GAPDH.

**Table 1:** Primers for real-time PCR

Gene Name	Primer sequences (5′–3′)	Accession no	Product size(bp)
*Occludin*	F: 5′-ACAGCCCTCAATACCAGGATGTG-3′ R: 5′-ACCATGCGCTTGATGTGGAA-3′	NC_205128.1	133
*Claudin-1*	F: 5′-ACACTACAATGCCGCTGTCCTGA-3′ R: 5′-CAATCTTTCCAGTGGCGATACCTAC-3′	NC_001013611.2	178
*Bdnf*	F: 5′-TTACCTGGATGCCGCAAACAT-3′ R: 5′-TGACCCACTCGCTAATACTGTC-3′	NC_000068.8	101
*Muc2*	F: 5′-AGGGCTCGGAACTCCAGAAA-3′ R: 5′-CCAGGGAATCGGTAGACATCG-3′	NC_28865873a1	106
*Zo-1*	F: 5′-GCCGCTAAGAGCACAGCAA-3′ R: 5′-TCCCCACTCTGAAAATGAGGA-3′	NC_6678355a1	134
*Lc3b*	F: 5′-TTATAGAGCGATACAAGGGGGAG-3′ R: 5′-CGCCGTCTGATTATCTTGATGAG-3′	NC_13385664a1	109
*P62*	F: 5′-AGGATGGGGACTTGGTTGC-3′ R: 5′-TCACAGATCACATTGGGGTGC-3′	NC_26324858a1	178
*Bcl-2*	F: 5′-ATGCCTTTGTGGAACTATATGGC-3′ R: 5′-CAATCTTTCCAGTGGCGATACCTAC-3′	NC_6753168a1	120
*Psd95*	F: 5′-TCCGGGAGGTGACCCATTC-3′ R: 5′-TTTCCGGCGCATGACGTAG-3′	NC_000077.7	83
*Syn*	F: 5′-CAGTTCCGGGTGGTCAAGG-3′ R: 5′-ACTCTCCGTCTTGTTGGCAC-3′	NC_6678195a1	138
*Mao-a*	F: 5′-GCCCAGTATCACAGGCCAC-3′ R: 5′-CGGGCTTCCAGAACCAAGA-3′	NC_27804325a1	117
*Gapdh*	F: 5′-CCGAGAATGGGAAGCTTGTC-3′ R: 5′-TTCTCGTGGTTCACACCCATC-3′	NC_000072.7	232

### Western blotting

Tissues from the colon and hippocampus were homogenized in radio immune precipitation assay (RIPA) lysis buffer (CW2333, CWBioTech, Beijing, China) containing 1 μL of protease inhibitor cocktail per sample (CW2200, CWBioTech, Beijing, China). After centrifugation at 12,000×*g* for 10 min at 4 °C, the supernatant was collected, and the protein concentration was determined using a bicinchoninic acid (BCA) protein assay kit (CW0014, CWBioTech, Beijing, China). The total protein was electrophoresed on a 4% stacking gel at 60 V for 60 min and then on an 8–12% separating gel at 100 V for 150 min. Then, the proteins were transferred to polyvinylidene fluoride (PVDF) membranes (Millipore, USA) at 200 mA for 90 min. PVDF membranes were first blocked with 5% skim milk for 1.5 h and then probed with specific primary antibodies (β-actin, 1:2000; Claudin-1, 1:500; Occludin, 1:2000; ZO-1, 1:1000; MUC2, 1:1000; ATG5, 1:1000; ATG7, 1:1000; P62, 1:1000; LC3, 1:1000; Beclin1, 1:1000; p-PI3K, 1:1000; p-AKT, 1:1000; PSD95, 1:1000; BDNF, 1:1000; Abcam, Cambridge, MA, USA), followed by a secondary antibody [goat anti-rabbit IgG, HRP conjugated (1:3000, CW0103; CWBioTech, Beijing, China)]. An enhanced chemiluminescence western blot kit (CW0049A, CWBioTech, Beijing, China) was used to visualize the bands according to the manufacturer’s instructions. The intensities of the bands were analysed using a Gel-Pro Analyzer 4.5 (Media Cybernetics, USA). The data are presented as the integrated optical density (IOD) of the bands/the IOD of the corresponding β-actin bands.

### Measurements of antioxidant activity and lipid peroxidation

Portions of the colonic segments were rapidly homogenized, and clarified lysates were obtained by centrifugation (200×*g* for 10 min) at 4 °C. The tissue extracts were stored at − 80 °C for antioxidant activity analysis. Five commercial kits (Nanjing Jiancheng Co. Ltd., Nanjing, China) were used to assay the activities of glutathione peroxidase (GSH-Px), malondialdehyde (MDA), superoxide dismutase (SOD), total antioxidant capability (T-AOC) and catalase (CAT) and the content using colorimetric methods. Each sample was assayed three times.

### Microbial sequencing

The contents of the mouse colon were collected and stored in liquid nitrogen. The MN Nucleo Spin 96 Soi DNA Extraction Kit (MACHEREY–NAGEL GmbH & Co. KG, Duren, Germany) was used to extract total bacterial DNA from the sample. The primers were designed according to the conserved region of microorganism V3 + V4. The primers were used for PCR amplification, and the products were purified, quantified and homogenized to form a sequencing library. The built library was first subjected to quality inspection, and the qualified library was sequenced with a NovaSeq 6000 (Illumina, Co., Inc., California, USA). Sequences with similarity greater than 97% were classified as operational taxonomic units (OTUs). Principal component analysis (PCA)-based principal coordinate analysis (PCoA) and nonmetric multidimensional scaling (NMDS) were used to analyse the OTU composition of different Bray‒Curtis samples. Line discriminant analysis (LDA) effect size (LEfSe) was used to analyse the significance of differences between groups from the phylum to genus level. LEfSe analysis required an LDA score > 4.

### Statistical analysis

The data involved in the study are all expressed as the mean ± SEM. The statistical analyses were performed via one-way analysis of variance (ANOVA) using IBM SPSS software (version 19.0, SPSS Inc., Chicago, IL), followed by Duncan’s multiple range test.

## Results

### CUMS accelerated more weight loss and aggravated depressed behaviour in female mice compared to male mice

**Fig. 1:**
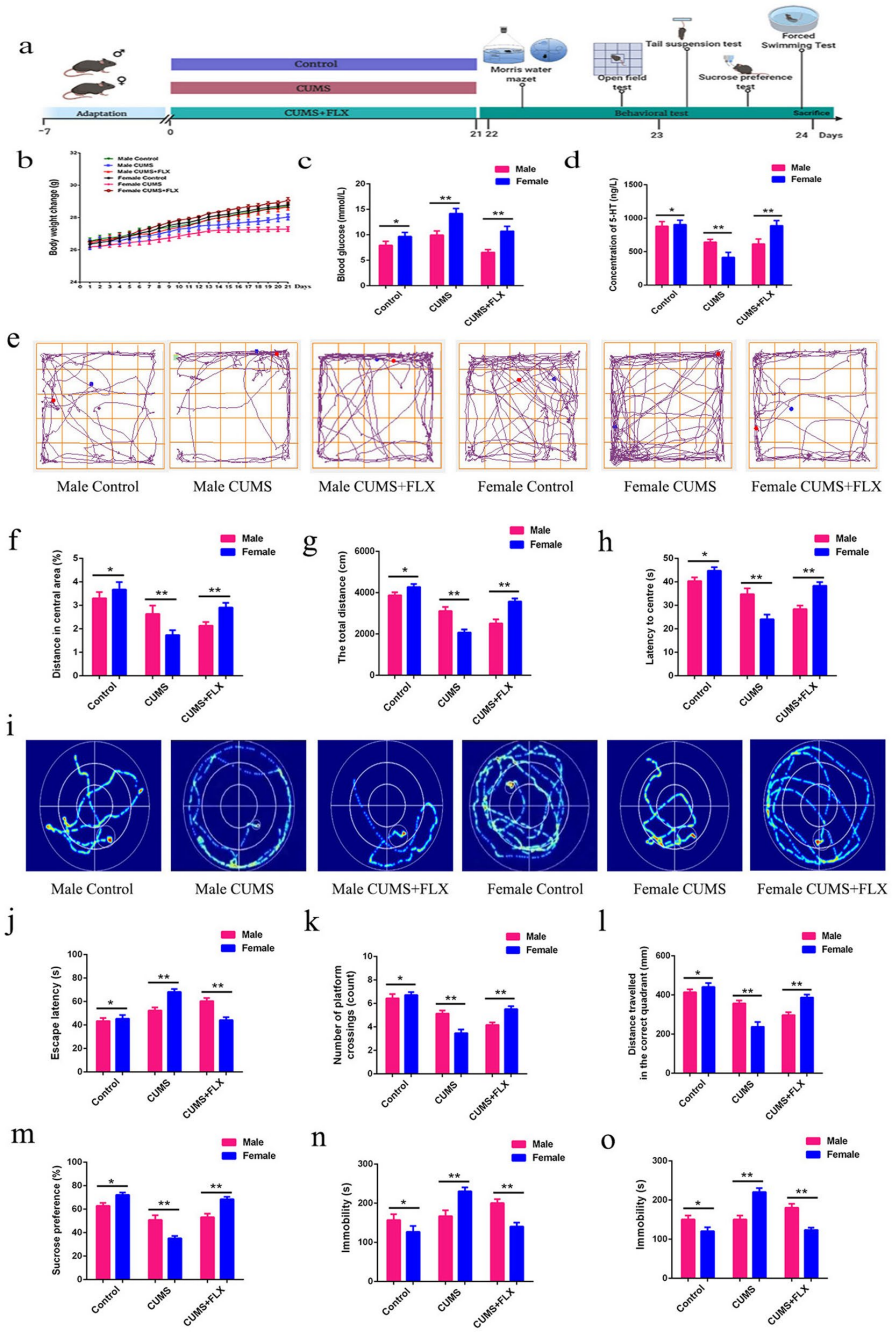
CUMS affects weight gain and depression-like behavior in both female and male mice. **a** Experimental design diagram. **b** Changes in body weight of mice. **c** Changes in blood sugar. **d** Content of 5-HT in hippocampus. **e** Road map of the open field experiment. **f** Ratio of distance between center and periphery. **g** Length of the total journey. **h** Center to peripheral delay time. **i** Path map of the morris water maze. **j** Escape latency. **k** Number of platform crossings. **l** Distance traveled in the correct quadrant. **m** Sugar water preference. **n** Forced to swim immobile time. **o** Tail suspension experiment immobility time. Each value represents the mean ± SEM. *p < 0.05, **p < 0.01 and ***p < 0.001 means difference of female vs. male mice at the same point

To explore the effect of CUMS on male and female mice, the following experiments with wild-type male and female mice groups (*n* = 10) were designed (Fig. 1a). From the effect of CUMS on body weight, it can be seen that CUMS decreased female mouse weight by 33.4% (*p* < 0.01) (Fig. 1b) and increased blood sugar by 35.3% (*p* < 0.01) (Fig. 1c), and after FLX treatment, the 5-HT content in the hippocampus was significantly increased by 40.2% (*p* < 0.01) (Fig. 1d). Further behavioural experiments showed that the ratio of the periphery to the centre and the length of the total distance in the treatment group were significantly higher than those in the stress groups by 36.9–48.7% (*p* < 0.01) (Fig. 1e–h). A clear lack of consciousness, for the water maze experiment, the latency was less than 30.5–46.1% of the female CUMS group than in the male CUMS group (*p* < 0.01) (Fig. 1i–l). The sugar water preference rates, tail-hanging test and forced swimming immobility time of the female CUMS group were reduced to a greater extent than those of the male CUMS group by 31.7% (*p* < 0.01), 35.6% (*p* < 0.01) and 42.5% (*p* < 0.01), respectively (Fig. 1m–o). The data suggested that the effect of CUMS on the behaviour of females was greater than that on males. Considering that BDNF plays a critical role in the pathophysiology of depression and is closely related to activated microglia in the hippocampus, we measured hippocampal protein and mRNA levels of BDNF and its high-affinity receptor TrkB to determine whether hippocampal BDNF contributes to sex differences in depressive-like behaviour. The quantity of BDNF protein was decreased in both sex CUMS groups. However, there was a significant difference in the quantity of BDNF protein between sexes. Specifically, the quantity of BDNF protein was 28.6% lower in female mice than in male mice (*p* < 0.01) (Additional file 1: Fig. S1a–c). The BDNF mRNA levels were consistent with the western blot results. In addition, the protein and mRNA expression levels of TrkB showed the same tendency as those of BDNF. However, the decrease in TrkB was 38.4% lower in female mice than in males (*p* < 0.01) (Additional file 1: Fig. S1d–e), and the CORT, NE, DA and iNOS mRNA levels decreased in both male and female mice in response to CUMS. However, the decrease in female mice was greater than that in male mice (40.1% (*p* < 0.01), 33.9% (*p* < 0.01), 30.6% (*p* < 0.01) and 38.4% (*p* < 0.01), respectively) (Additional file 1: Fig. S1f–i). The results showed that CUMS had more significant effects on BDNF and TrKB proteins and related neurotransmitters in the hippocampi of female mice. The data suggested that the effect of CUMS on the behaviour of females was greater than that on males.

### 5-HT attenuated depressive symptoms in mice exposed to CUMS

**Fig. 2:**
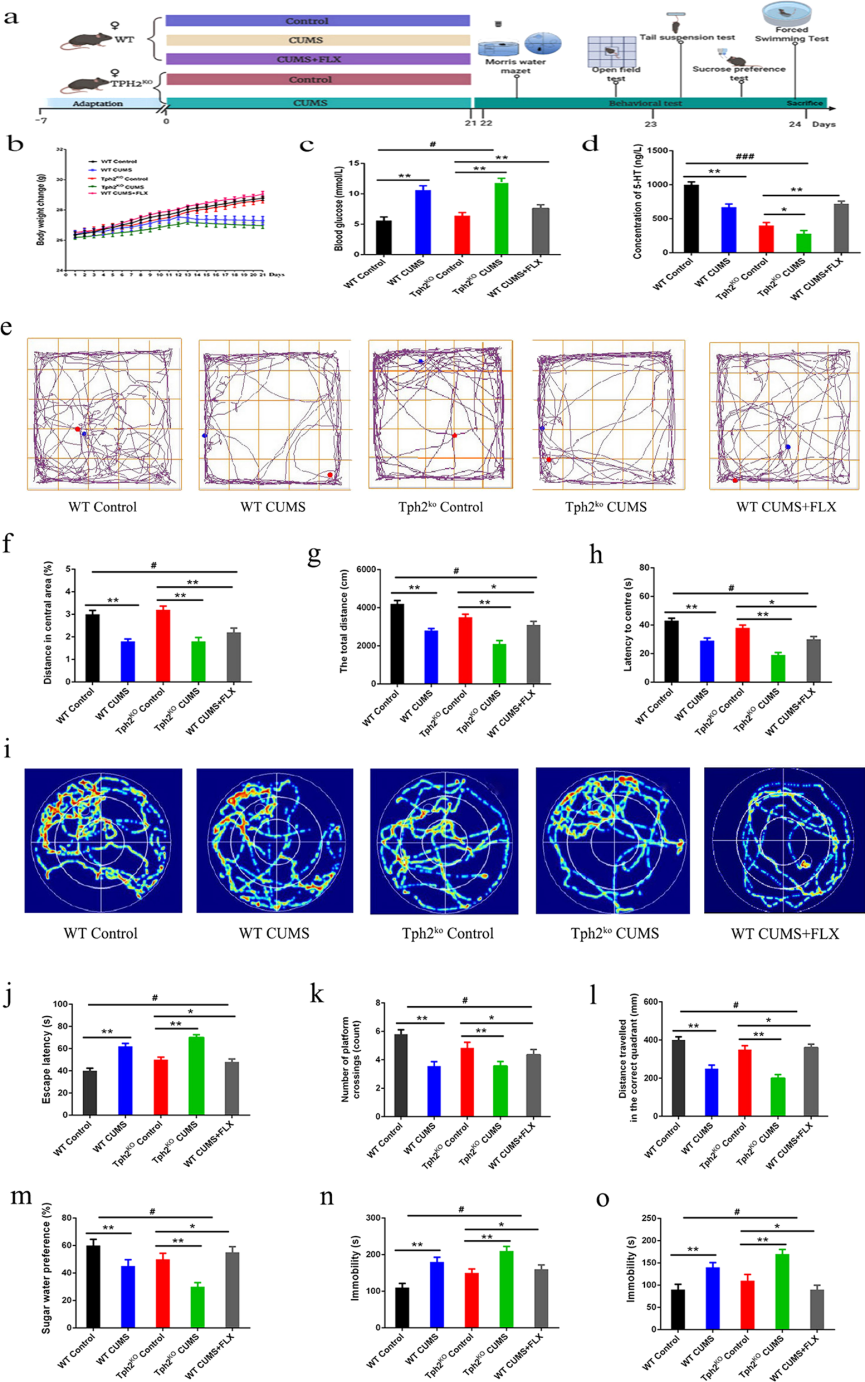
Depressive symptoms mice exposed to CUMS. **a** Experimental design diagram. **b** Changes in body weight of mice. **c** Changes in blood sugar. **d** Content of 5-HT in hippocampus. **e** Road map of the open field experiment. **f** Ratio of distance between center and periphery. **g** Length of the total journey. **h** Center to peripheral delay time. **i** Path map of the morris water maze. **j** Escape latency. **k** Number of platform crossings. **l** Distance traveled in the correct quadrant. **m** Sugar water preference. **n** Forced to swim immobile time. **o** Tail suspension experiment immobility time. Each value represents the mean ± SEM. *p < 0.05, **p < 0.01 and ***p < 0.001 means difference of WT Control vs. WT CUMS, TPH2 KO vs. TPH2 KO CUMS, TPH2 KO vs. WT CUMS + FLX at the same point

Based on the above data, female mice were used for the following tests. To explore the effect of 5-HT on CUMS mice, the following experiments were designed with five groups, (1) WT control group, (2) WT CUMS group, (3) WT CUMS + FLX (20 mg/kg) group, (4) TPH2 KO group, and (5) TPH2 KO CUMS group (*n* = 10) (Fig. 2a). Compared with the WT CUMS group, the TPH2 KO CUMS group had reduced body weight (39.5%, *p* < 0.01) (Fig. 2b), increased blood sugar (36.7%, *p* < 0.01) (Fig. 2c), and decreased content of 5-HT in the hippocampus. FLX increased the 5-HT content in the hippocampus by 42.6% (*p* < 0.01) (Fig. 2d). Further behavioural experiments showed that the results of the open field test, the ratio of the periphery to the centre and the length of the total distance in the FLX treatment group were significantly higher by 32.5–45.6% (*p* < 0.01) than those in the TPH2 KO CUMS group (Fig. 2e–h). Compared with the WT CUMS group, the delay of the water maze test in the TPH2 KO CUMS group was higher by 33.8–46.8% (*p* < 0.01) (Fig. 2i–l). Sugar water preference rates, tail-hanging tests and forced swimming immobility time were reduced by 42.5% (*p* < 0.01), 30.5% (*p* < 0.01) and 36.4% (*p* < 0.01), respectively. FLX treatment increased WT CUMS-induced 5-HT concentrations, thereby alleviating learning and memory impaired behaviours (Fig. 2m–o). These results suggest that 5-HT can improve learning and memory behaviour of mice. Data Set GSE151807 in the GEO database was selected, which contained 12 mouse hippocampal tissue samples, including 6 mice in the control group and 6 mice in the depression group. The results showed that compared with control mice, the expression levels of 351 genes in the hippocampus of depressed mice were changed, of which 182 genes were upregulated and 169 genes were downregulated, as shown in Additional file 2: Fig. S2a. The top 10 genes (BDNF, PSD95, SYN, INIP, BTBD18, FNTB, TSRR2, TMEM101, NOP14 and NFAT5) with significant differences in upregulation and downregulation were selected to construct a heatmap to display changes in DEG expression (Additional file 2: Fig. S2b). GSEA of the GSE151807 gene expression matrix showed that genes related to neuroactive ligand‒receptor interactions, ubiquitin-mediated proteolysis, Huntington's disease, glutathione metabolism, peroxidase, and long-term depression were significantly enriched in the hippocampus and amygdala tissues of depressed mice, as shown in Additional file 2: Fig. S2c–h. KEGG pathway analysis showed that upregulated DEGs were obviously abundant in signalling pathways regulating pluripotency of stem cells, mTOR signalling pathway, Hippo signalling pathway, RNA degradation, hepatocellular carcinoma, adrenergic signalling in PI3K–Akt signalling pathway, melanogenesis pathway and other pathways (Additional file 2: Fig. S2i). These results suggest that 5-HT can improve the learning and memory behaviour of mice.

### 5-HT on five antioxidant parameters (GSH-Px, MDA and SOD, T-AOC and CAT) in CUMS mice

**Fig. 3:**
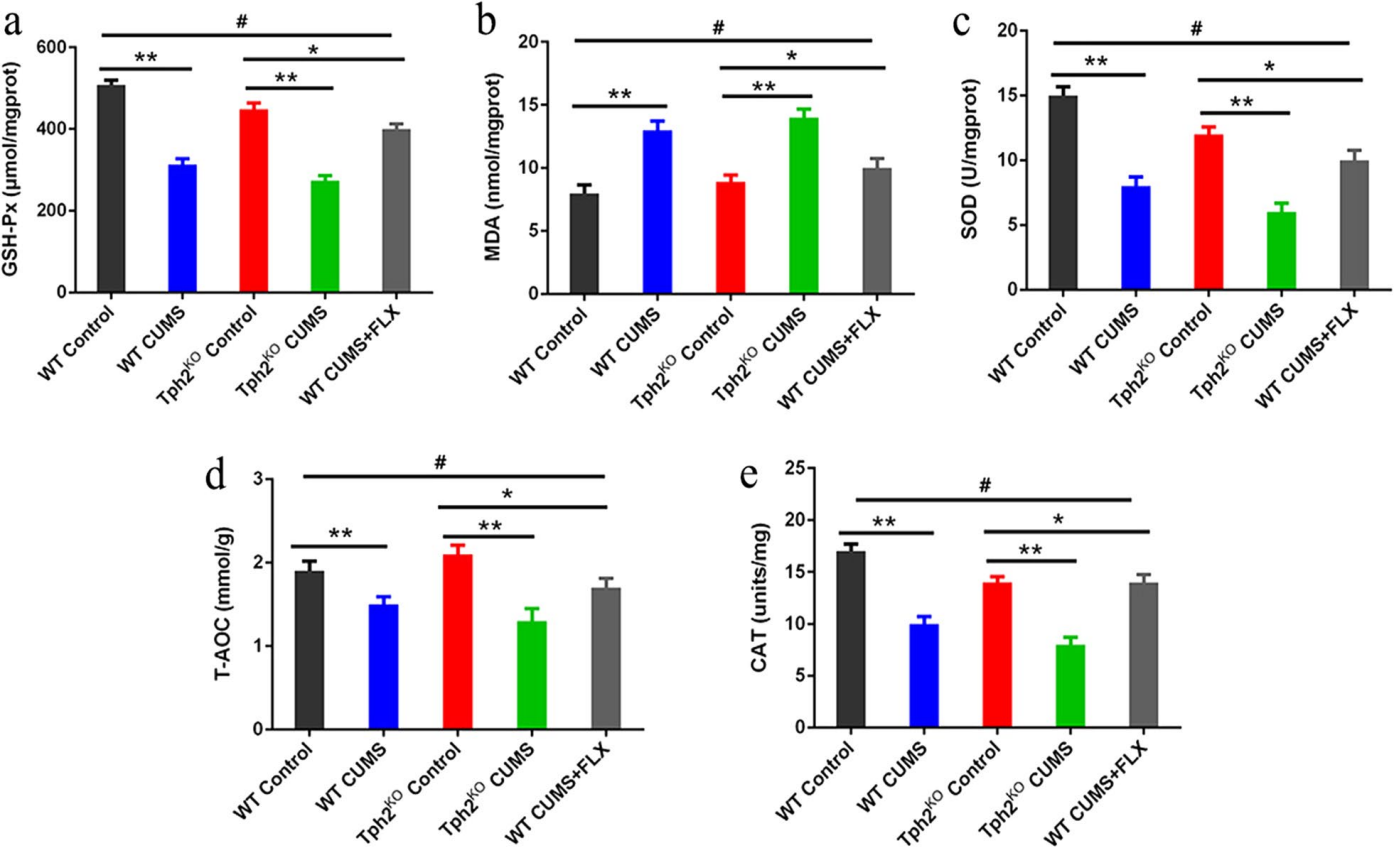
Effects of CUMS five antioxidant indices in hippocampus of mice. **a** GSH-Px. **b** MDA. **c** SOD. **d** T-AOC. **e** CAT concentrations in the hippocampus were measured using an oxidative stress-related enzyme test. Each value represents the mean ± SEM. *p < 0.05, **p < 0.01 and ***p < 0.001 means difference of WT Control vs. WT CUMS, TPH2 KO vs. TPH2 KO CUMS, TPH2 KO vs. WT CUMS + FLX at the same point

We then examined whether CUMS caused oxidative stress in the hippocampus by examining five antioxidant parameters, including antioxidant enzymes (GSH-Px, SOD and CAT), T-AOC and MDA, in the hippocampus (Fig. 3). Compared with the WT control group, the WT CUMS group had significantly higher levels of MDA (36.7%, *p* < 0.01) and significantly reduced levels of GSH-Px (38.6%, *p* < 0.05), SOD (40.8%, *p* < 0.01), T-AOC (39.8%, *p* < 0.05) and CAT (40.8%,* p* < 0.01). However, compared with the TPH2 KO group, the TPH2 KO CUMS group had significantly higher levels of MDA (46.3%, *p* < 0.01) and significantly reduced levels of GSH-Px (47.4%, *p* < 0.05), SOD (33.3%, *p* < 0.01), T-AOC (56.7%, *p* < 0.01) and CAT (36.8%,* p* < 0.01). After FLX treatment, the WT CUMS + FLX group had significantly reduced levels of MDA (37.5%, *p* < 0.01) and increased levels of GSH-Px (40.4%, *p* < 0.01), SOD (42.7%, *p* < 0.01), T-AOC (46.1%, *p* < 0.01) and CAT (46.3%,* p* < 0.01). These results indicated that the antioxidant capacity was significantly decreased in the hippocampus after CUMS and that increased 5-HT content could reverse the oxidative stress damage caused by CUMS.

### 5-HT promotes neurogenesis in the hippocampus of CUMS mice

**Fig. 4:**
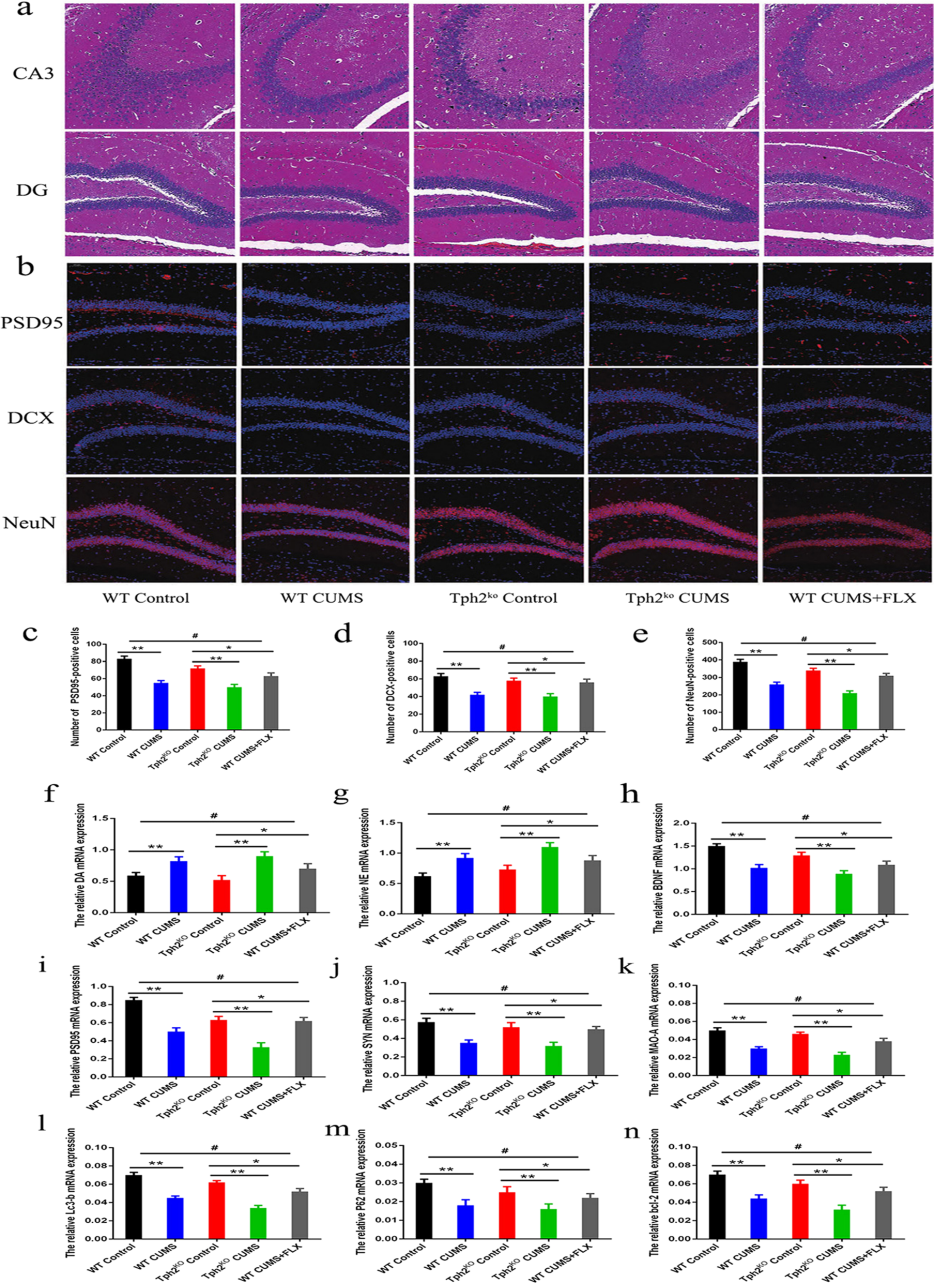
CUMS mice reduced hippocampal neurogenesis. **a** Comparison of microstructures of hippocampal CA3 and DG regions of mice in each group. **b** Immunofluorescence of PSD95, DCX and NeuN in hippocampal DG region of mice in each group. **c** Statistics on the number of PSD95 positive cells in the hippocampal DG area. **d** Statistics on the number of DCX positive cells in the hippocampal DG area. **e** Statistics on the number of NeuN positive cells in the hippocampal DG area. **f** mRNA levels of the DA expression in hippocampus. **g** mRNA levels of the NE expression in hippocampus. **h** mRNA levels of the BDNF expression in hippocampus. **i** mRNA levels of the PSD95 expression in hippocampus. **j** mRNA levels of the SYN expression in hippocampus. **k** mRNA levels of the MAO-A expression in hippocampus. **l** mRNA levels of the Lc3b expression in hippocampus. **m** mRNA levels of the p62 expression in hippocampus. **n** mRNA levels of the bcl-2 expression in hippocampus. Each value represents the mean ± SEM. *p < 0.05, **p < 0.01 and ***p < 0.001 means difference of WT Control vs. WT CUMS, TPH2 KO vs. TPH2 KO CUMS, TPH2 KO vs. WT CUMS + FLX at the same point

To further investigate whether CUMS caused damage to the hippocampus, we conducted statistical analysis of the structure of the hippocampal CA3 and DG. The cells in the CA3 and DG areas in the hippocampus of the control group were neatly arranged, the cell structure was clear, the staining was uniform, and the cells were tightly connected. Compared with the WT CUMS group, in the TPH2 KO CUMS group, the intercellular spaces in the CA3 and DG regions of the hippocampus increased, the cells were not arranged neatly, the nuclei were more pyknotic, and the number of cells was significantly reduced. In the FLX treatment group, the cells in the hippocampal CA3 and DG areas of the mice were arranged slightly neatly, and nuclear pyknosis was reduced (Fig. 4a). PSD95 marks the postsynaptic density of neurons, DCX marks immature neurons and NeuN marks mature neurons. In the figure, blue is the nucleus, and red is the target protein (Fig. 4b). Compared with the WT CUMS group, the number of PSD95-, DCX-, and NeuN-positive cells in the hippocampal DG in the TPH2 KO CUMS groups was significantly reduced by 46.8% (*p* < 0.01). In addition, in the FLX-treated WT CUMS group, the number of positive cells labelled with PSD95, DCX, and NeuN in the region were increased 38.45% (*p* < 0.01), 32.67% (*p* < 0.01), 40.21% (*p* < 0.01) and 36.69% (*p* < 0.01) compared with the WT CUMS group (Fig. 4c–e). More specifically, the mRNA levels of DA, NE and BDNF were significantly increased by 46.36% (*p* < 0.01), 43.82% (*p* < 0.01) and 47.35% (*p* < 0.01), respectively, in the FLX treatment group compared to the TPH2 KO CUMS group. The synapse-related mRNA levels of SYN, PSD95 and MAO-A and the autophagy-related gene mRNA levels of bcl-2, lc3b and p62 were increased by 35.36% (*p* < 0.01), 40.12% (*p* < 0.01), 36.52% (*p* < 0.01), 38.12% (*p* < 0.01), 40.35% (*p* < 0.01) and 38.56%, respectively (Fig. 4f–n). These results suggest that 5-HT can alleviate CUMS-induced hippocampal structural damage.

### 5-HT suppresses CUMS-induced autophagy and mediates PI3K/Akt/mTOR pathway impairment in the hippocampus

**Fig. 5:**
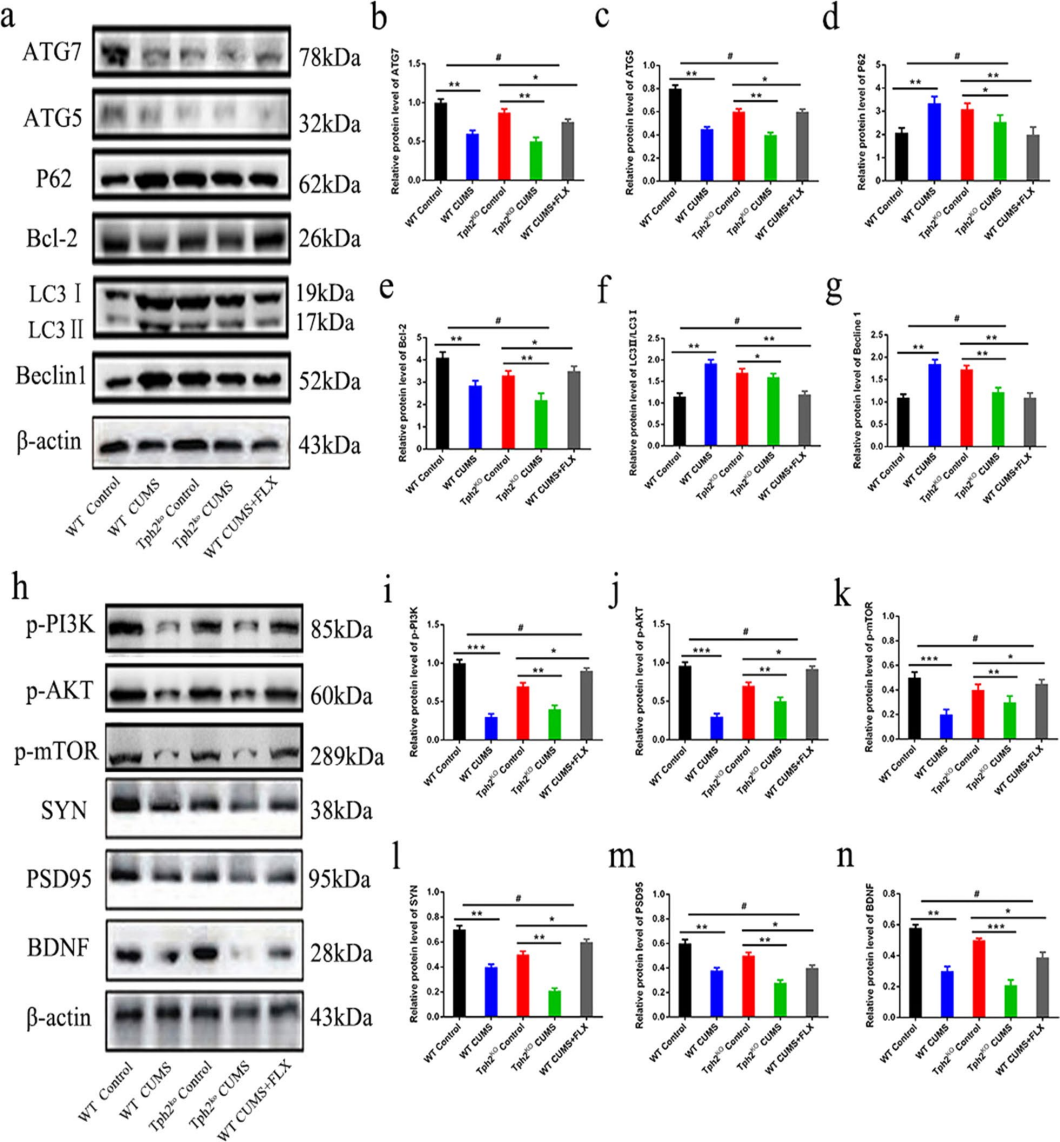
Effects of CUMS on PI3K/Akt/mTOR and autophagy pathway in mice. **a** Electrophoretogram of ATG7, ATG5, P62, Bcl-2, Lc3, Beclin1 and β-actin protein in hippocampus by western blotting. **b** Data of relative expression of ATG7 protein. **c** Data of relative expression of ATG5 protein. **d** Data of relative expression of P62 protein. **e** Data of relative expression of Bcl-2 protein. **f** Data of relative expression of LC3 protein. **g** Data of relative expression of Beclin1 protein. **h** Electrophoretogram of p-PI3K, p-AKT, p-mTOR, SYN, PSD95, BDNF and β-actin protein in hippocampus by western blotting. **i** Data of relative expression of p-PI3K protein. **j** Data of relative expression of p-AKT protein. **k** Data of relative expression of p-mTOR protein. **l** Data of relative expression of SYN protein. **m** Data of relative expression of PSD95 protein. **n** Data of relative expression of BDNF protein. Each value represents the mean ± SEM. *p < 0.05, **p < 0.01 and ***p < 0.001 means difference of WT Control vs. WT CUMS, TPH2 KO vs. TPH2 KO CUMS, TPH2 KO vs. WT CUMS + FLX at the same point

The western blot assay revealed that p62 and LC3b levels in the CUMS group were significantly increased compared with those in the control group, while p62, LC3b, ATG7 and ATG5 (the upstream proteins of the autophagy pathway that are involved in the maturation of LC3b) were significantly decreased by 32.4–44.2% (*p* < 0.01) in the FLX treatment group (Fig. 5a–g). These data suggested that along with neuroinflammation, CUMS mediated dysregulated autophagy in the hippocampus, but FLX treatment could reverse these changes. On the other hand, the TPH2 KO CUMS group exhibited a significant decrease in phosphorylated PI3K, phosphorylated Akt, phosphorylated mTOR, PSD95, SYN and BDNF expression in the hippocampus compared with WT CUMS mice. However, varying degrees of increases of 30.6–42.6% (*p* < 0.01) were observed in the expression of PSD95, SYN, BDNF, phosphorylated p-PI3K, p-Akt, and p-mTOR in all FLX-treated groups (Fig. 5h–n). These results indicated that 5-HT plays an important role in the CUMS-regulated PI3K/Akt/mTOR and autophagy pathways.

### 5-HT ameliorates the disruption of gut tight junctions and colonic inflammation in mice induced by CUMS

**Fig. 6:**
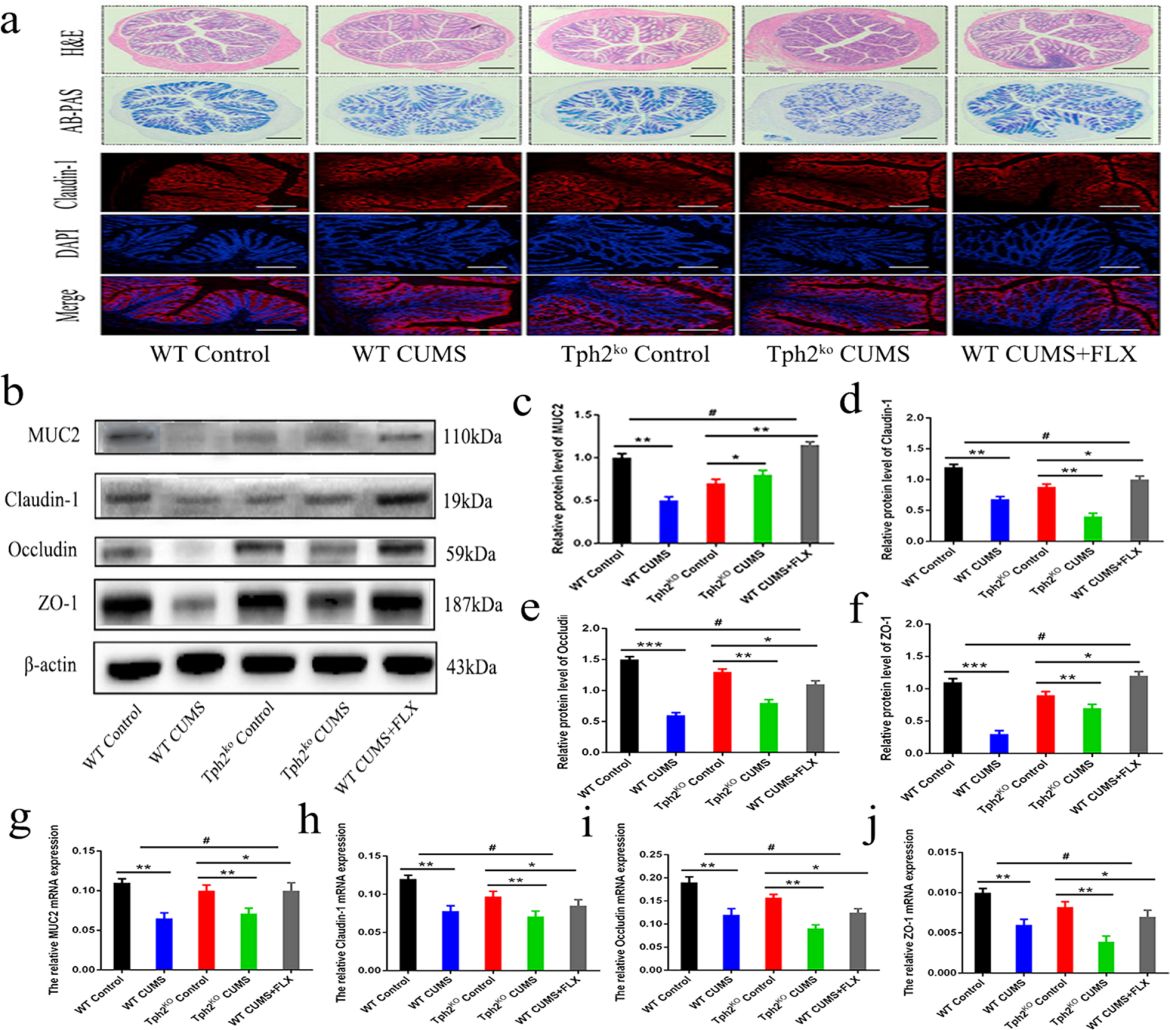
Increased inflammation and reduced tight junctions of the colon in CUMS mice. **a** Immunofluorescence assay for Claudin-1 (red) and DAPI (blue) expression in the colon of mice (× 40 magnifications). Morphological changes in the colon, as seen with H&E and AB-PAS staining (× 40 magnifications). Scale bar: 200 μm. **b**–**f** Western blotting analysis and quantification data of MUC2, Claudin-1, Occludin and ZO-1 in the colon of mice. **g**–**j** mRNA expression of MUC2, Claudin-1, Occludin and ZO-1 in the colon of mice. Each value represents the mean ± SEM. *p < 0.05, **p < 0.01 and ***p < 0.001 means difference of WT Control vs. WT CUMS, TPH2 KO vs. TPH2 KO CUMS, TPH2 KO vs. WT CUMS + FLX at the same point

The microbiota–gut–brain axis plays an important role in the development of depression. Next, we further evaluated the effect of CUMS on intestinal barrier function. Immunofluorescence staining of the colon was performed. H&E staining indicated indentations, cellular damage, and extensive separation of glands in the colonic epithelium of mice after CUMS exacerbated the control mice, which was associated with decreased mucus secretion. FLX relieved these symptoms compared to the CUMS group (Fig. 6a). Meanwhile, the results revealed that CUMS markedly reduced the level of claudin-1 in the colon of TPH2 KO mice. Consistent with the colon histology results, the protein levels of MUC2, claudin-1, occludin and ZO-1 were increased by FLX treatment by 28.6–41.32% (*p* < 0.01) in the TPH2 KO CUMS group (Fig. 6b–f). The relative mRNA levels of tight junction- and mucin-related markers, including MUC2, claudin-1, occludin and ZO-1, were also increased by 36.3–46.3% by FLX treatment (*p* < 0.01) (Fig. 6g–j). These results suggest that 5-HT may ameliorate the destruction of the intestinal tight junction barrier and colon inflammation induced by CUMS.

### The effect of 5-HT on gut microbiota composition disorder induced by CUMS

**Fig. 7:**
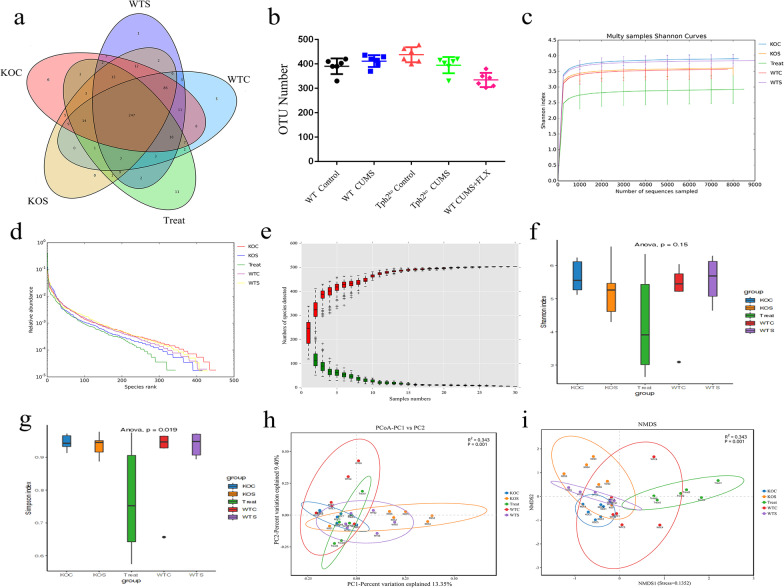
Venn diagram of colonic microorganisms OTU number, α diversity and β diversity responses in the colon of CUMS mice. **a** Venn diagram showing the number of OTUs in the WT Control, WT CUMS,TPH2 KO Control,TPH2 KO CUMS and WT CUMS + FLX groups. **b** Statistics for the four groups. The α diversity includes diversity and richness. **c** Shannon curves, **d** OTU rank curves, **e** Rank abundance curve, **f** Shannon index, **g** Simpson index of WT Control, WT CUMS, TPH2 KO Control, TPH2 KO CUMS and WT CUMS + FLX. The β diversity shows the dispersion of each sample in the WT Control, WT CUMS, TPH2 KO Control, TPH2 KO CUMS and WT CUMS + FLX groups. **h** Principal component analysis (PCA), **i** Nonmetric multidimensional scaling (NMDS) score plot based on the Bray–Curtis score plot based on the OTU in the colon. Values are presented as the means ± SEM. WTC: WT Control, WTS: WT CUMS, KOC: TPH2 KO Control, KOS: TPH2 KO CUMS, Treat: WT CUMS + FLX

**Fig. 8:**
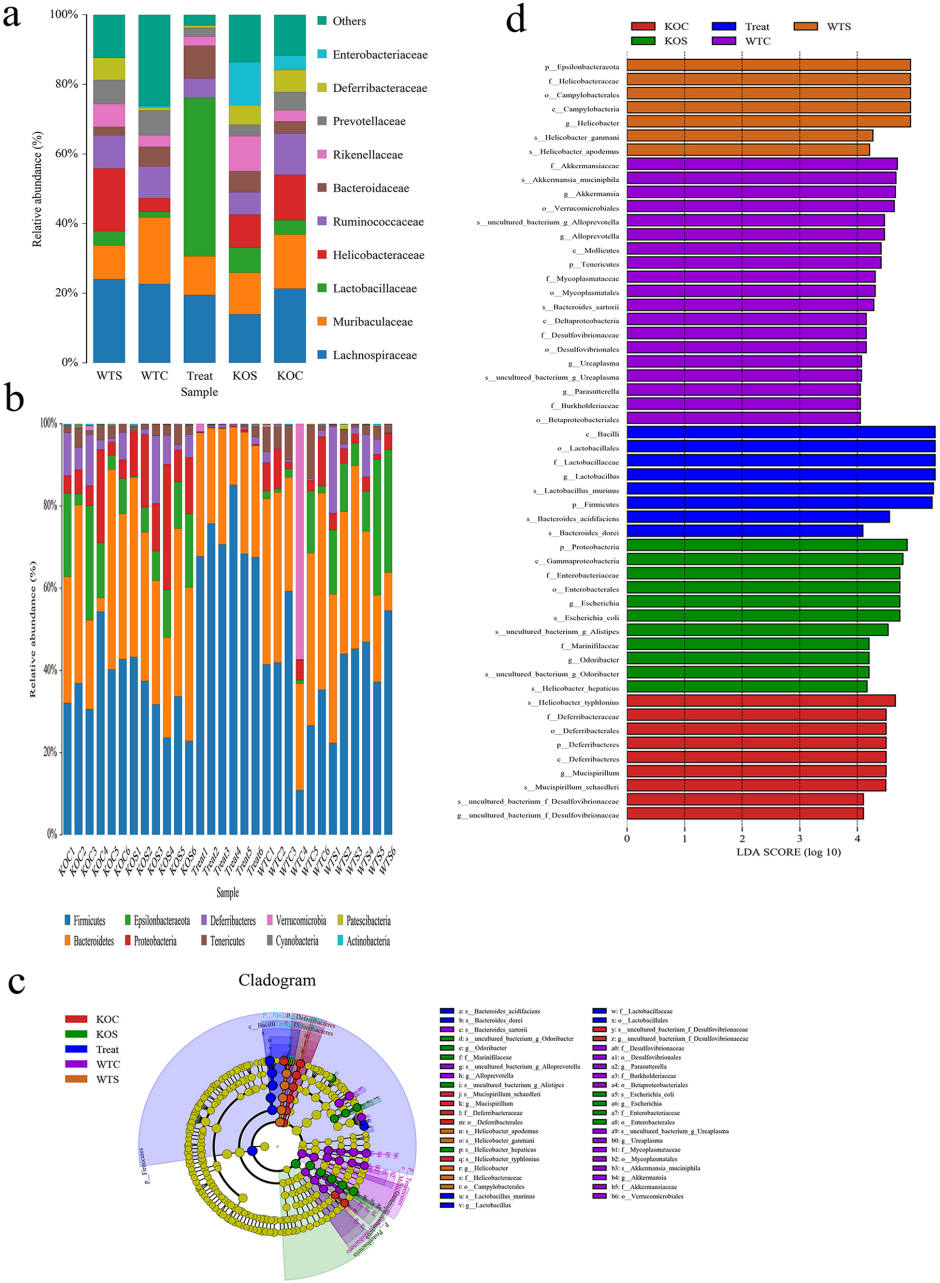
Composition of the colon microbiota. Relative contribution of the top 10 phyla in the WT Control, WT CUMS, TPH2 KO Control, TPH2 KO CUMS and WT CUMS + FLX groups in the colon. **a** Relative abundance of the top 22 genera in the Control, WT CUMS, TPH2 KO Control, TPH2 KO CUMS and WT CUMS + FLX groups in the colon. **b** Taxonomic cladogram obtained from LEfSe sequence analysis. **c** In the colon biomarker taxa are highlighted by coloured circles and shaded areas. The diameter of each circle reflects the abundance of that taxa in the community. **d** Taxa with a different abundance in the colon (*n* = 6). A cutoff value ≥ 2.0 was used for the linear discriminant analysis (LDA). WTC: WT Control, WTS: WT CUMS, KOC: TPH2 KO Control, KOS: TPH2 KO CUMS, Treat: WT CUMS + FLX

Next, we assessed whether CUMS and 5-HT could affect the gut microbiota. 16S rRNA high-throughput pyrosequencing showed that based on the 97% similarity level, all the effective reads were clustered into OTUs. Among the five treatment groups, including the WT Control, WT CUMS, TPH2 KO Control, TPH2 KO CUMS and WT CUMS + FLX groups, the OTU numbers were not significantly different (Fig. 7a–b). However, the Shannon curves, OTU rank curves, rank abundance curve, Shannon index, and Simpson index analysis showed that the richness and diversity of the colonic microbiota were significantly decreased in the TPH2 KO CUMS group. Compared with the CUMS group, quantitative perspective analysis showed that the ACE, Chao and Shannon indices were significantly decreased by 21.5% (*p* < 0.01) and 18.9% (*p* < 0.01) and the Simpson index was markedly increased by 47.5% (*p* < 0.05) in the TPH2 KO CUMS group (Fig. 7c–g). The trend of all indices was similar to that in the control group, with no statistically significant difference between the control group and the FLX supplementation group. The β-diversity analysis presented a distinct clustering of the colonic microbiota composition in all groups (Fig. 7h–i). Specifically, the phylum and genus level analysis demonstrated that TPH2 KO CUMS significantly decreased the relative abundance of *Lactobacillus* and increased the relative abundance of *Proteobacteria,* while supplementation with FLX restored these levels (Fig. 8a, b). These results showed that CUMS suppressed the diversity and richness of the colonic microbiota but increased the abundance of *Mollicutes*, *Proteobacteria*, *Desulfovibrionales* and *Deltaproteobacteria*. These results suggested that 5-HT supplementation modulated the abundance and diversity of colon microorganisms induced by CUMS.

**Fig. 9:**
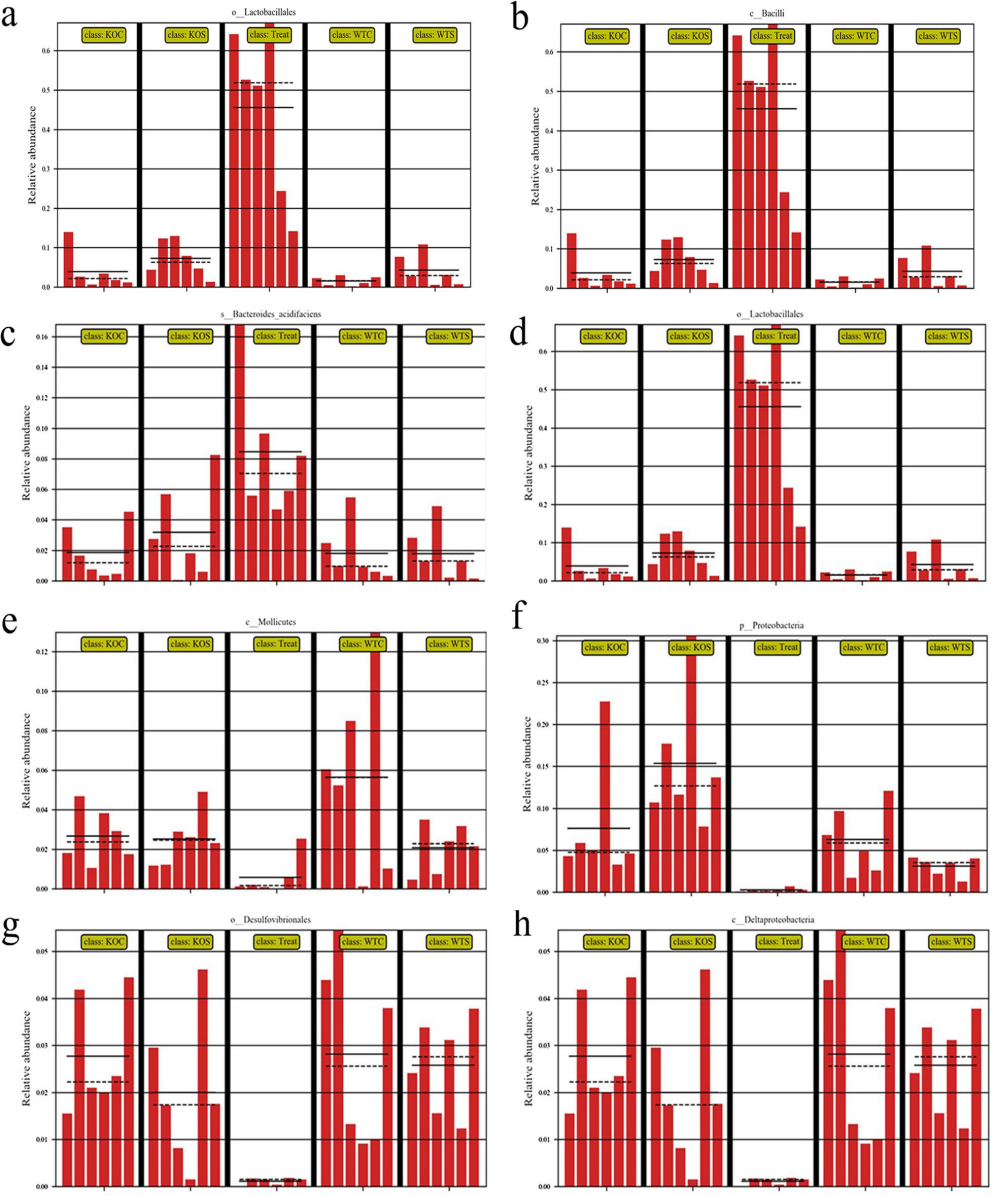
Comparison diagram of abundance of colon. **a** *Lactococcus.* **b** *Bacilli.* **c** *Bacteroides* and **d** *Lactobacillales*. **e** *Mollicutes.* **f** *Proteobacteria.* **g** *Desulfovibrionales* and **h** *Deltaproteobacteria* for significance analysis of differences between groups. The solid and dashed lines represent the average and median of the relative abundance in each group of samples. WTC: WT Control, WTS: WT CUMS, KOC: TPH2 KO Control, KOS: TPH2 KO CUMS, Treat: WT CUMS + FLX

To identify the specific bacterial taxa associated with CUMS and 5-HT supplementation, we employed the linear discriminant analysis (LDA) effect size (LEfSe) method. A cladogram representative of the colonic microbiota structure displayed the predominant bacteria and the greatest differences in taxa among the 5 communities (Fig. 8c, d). The results showed that the predominant bacteria in the colon of TPH2 KO CUMS mice were *Bacteroides, Bacilli* and *Lactobacillus.* FLX supplementation significantly suppressed these pathogenic bacteria, although the predominant bacteria were not exactly the same in the control group. We further analysed the effective sequences of all samples by redundancy analysis (RDA) to identify the phylotypes of the gut microbiota that responded to FLX supplementation. After supplementation with FLX, there were no differences compared with the control group. However, TPH2 KO CUMS mice significantly decreased the content of *Mollicutes*, *Proteobacteria* and *Desulfovibrionales,* while it increased the content of *Deltaproteobacteria* (Fig. 9). In contrast, supplementation with FLX induced a large increase in *Lactococcus, Bacilli* and *Lactobacillale*s and a decrease in *Bacteroides*. Collectively, these results indicated that CUMS exacerbated the intestinal flora disorder in TPH2 KO mice, and the intestinal flora disorder caused by CUMS was alleviated after FLX treatment, suggesting that 5-HT can alleviate the intestinal flora disorder caused by CUMS.

### *Lactococcus lactis* E001-B-8 attenuated depressive symptoms in mice exposed to CUMS

**Fig. 10:**
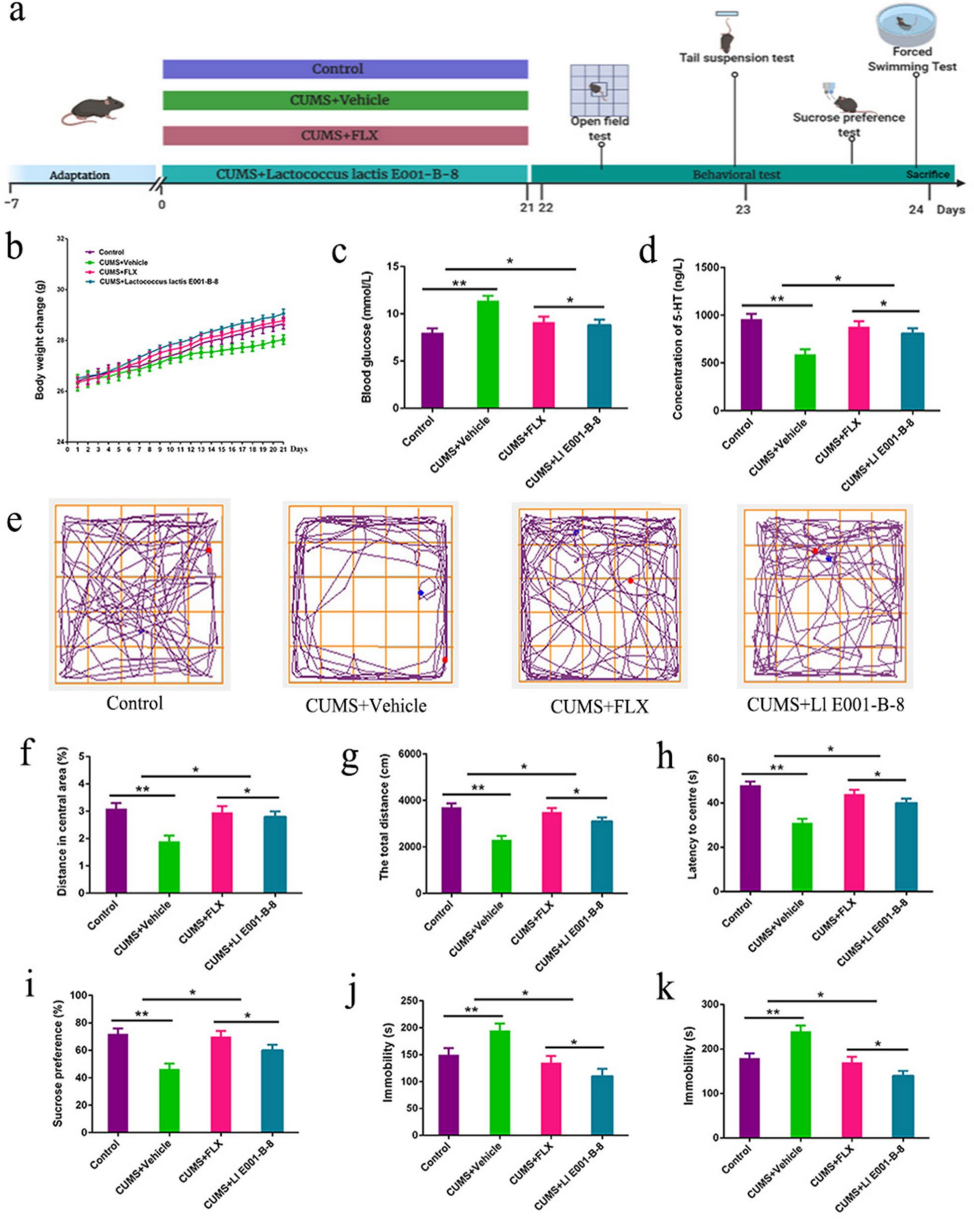
*Lactococcus lactis* E001-B-8 attenuated the depressive symptoms mice exposed to CUMS. **a** Experimental design diagram. **b** Changes in body weight of mice. **c** Changes in blood sugar. **d** Content of 5-HT in hippocampus. **e** Road map of the open field experiment. **f** Ratio of distance between center and periphery. **g** Length of the total journey. **h** Center to peripheral delay time. **i** Sugar water preference. **j** Forced to swim immobile time. **k** Tail suspension experiment immobility time. Each value represents the mean ± SEM. *p < 0.05, **p < 0.01 and ***p < 0.001 means difference of Control vs. CUMS + Vehicle, CUMS + FLX vs. CUMS + *Lactococcus lactis* E001-B-8 at the same point

According to the intestinal flora sequencing results, *Lactococcus* was most obvious in the CUMS and FLX treatment groups. *Lactococcus lactis* E001-B-8 is one of the *Lactococcus* species in our lab, and it was found to promote 5-HT production. To explore the effect of *Lactococcus lactis* E001-B-8 on CUMS mice, mice were divided into 4 different groups: (1) Control, (2) CUMS + Vehicle, (3) CUMS + FLX, and (4) CUMS + *Lactococcus lactis* E001-B-8 group (*n* = 10). The *Lactococcus lactis* E001-B-8 (1 × 10^9^ CFU mL^−1^) fungus powder was orally administered at 200 μL, and the mice were exposed to CUMS and orally administered Vehicle, FLX, or *Lactococcus lactis* E001-B-8 for 3 consecutive weeks. The following experiments were designed (Fig. 10a). Compared to the CUMS + Vehicle group, *Lactococcus lactis* E001-B-8 increased the body weight (*p* < 0.01) (Fig. 10b) of CUMS mice, relieved the increase in blood sugar induced by CUMS (*p* < 0.01) (Fig. 10c), and increased the 5-HT content in the hippocampus (*p* < 0.01) (Fig. 10d). From the results of the open field test, compared with the CUMS group, the ratio of the periphery to the centre and the length of the total distance were significantly increased by 36.9% (*p* < 0.01) and 48.7% (*p* < 0.01), respectively, in the CUMS + *Lactococcus lactis* E001-B-8 group (Fig. 10e–h). The sugar water preference rate and stationary jump time of the *Lactococcus lactis* E001-B-8 group were 31.7–42.5% lower than those of the CUMS group (*p* < 0.01) (Fig. 10i–k). These results were not significantly different between the CUMS + *Lactococcus lactis* E001-B-8 group and the FLX treatment group. The data suggested that *Lactococcus lactis* E001-B-8 can increase 5-HT content and has an effect on learning and memory in mice.

## Discussion

Depression is the main neuropsychiatric disorder affecting more than 320 million people worldwide (World Health Organization, 2017). It strongly increases the risk of suicide and affects the quality of life of individuals. Although the exact aetiology and pathophysiology of depression are still elusive, modern theories about the development of depression have involved monoaminergic system dysfunction, immune inflammation in the periphery and central nervous systems, and dysbiosis of the gastrointestinal microbiome [56]. GBA refers to the continuous bidirectional communication between the gastrointestinal tract and the brain. The main function of this system is to connect gut function with processes taking place in the central nervous system, including emotional and cognitive-type reactions [57]. 5-HT is also produced in enteric neurons, where it acts as a neurotransmitter. However, serotonin also has hormonal, autocrine, and paracrine actions and can act as a growth factor [58]. Most of the serotonin produced in the gastrointestinal tract is secreted into the systemic circulation and is subsequently stored in circulating platelets [59]. Stress affects endocrine effects and may, therefore, have inconsistent effects on sex. In the current study, CUMS differentially affected the behaviours of male and female mice. Compared with the male mice, female mice exhibited a slower pace of body weight gain and a faster reduction in relative weight when they were subjected to inescapable CUMS. In addition, the decreases in sucrose preference and central activity durations were greater in female mice than in males. These results indicated that different sexes led to different sensitivities to stress, and females tended to be more susceptible than males. This phenomenon is partly consistent with that from another study repeatedly injecting animals with lipopolysaccharide [60]. As a biomarker reflecting the activation of the HPA axis, corticosterone (CORT) has been widely used as a plausible parameter to evaluate stress levels in animals. Within this study, we first observed that the basal serum CORT increased in both sexes of CUMS mice. However, we noticed that the CORT level in the female CUMS group was elevated more than that in male mice after the CUMS procedure. These results coincided with previous studies involving animals and humans [61, 62]. Therefore, we may conclude that males tend to maintain relatively stable levels of hormones when faced with danger, while females show overactivated HPA axis functions under pressure [63]. It has become a hot research topic in life science and medical circles to study the antioxidant mechanism of antioxidants and find high-efficiency and low-toxicity antioxidants to better serve human and improve people's life quality. Antioxidants are a class of substances that can effectively prevent or delay automatic oxidation. Halliwell is defined as a substance that can effectively delay or prevent the oxidation reaction of the substrate at a lower concentration than that of the oxidizable substrate (sugar, lipid, DNA or protein) [64]. In living organisms, oxidation is always accompanied by the release of reactive oxygen species. The types, mechanisms, sites and targets of reactive oxygen species are different, and the types, mechanisms and abilities of antioxidants are also different. CUMS is an international common method to establish experimental animal depression models. This method induces excessive free radicals in animals and causes the imbalance of oxidation-antioxidant stress system, resulting in symptoms similar to those in patients with clinical depression [65]. Recently, it has been reported that patients with clinically severe depression have increased levels of free radicals in their blood and decreased total antioxidant capacity. The levels of free radicals and lipid peroxidation in the blood of the patients after antidepressant treatment decreased, suggesting that the mechanism of depression may be closely related to the oxidation-antioxidant stress system [66].

The gut–brain axis is generally considered to be involved in psychiatric diseases, and neurobiological aetiological factors underlying depression have been proposed, including deficits in other neurotransmitters and neurotrophic factors, such as brain-derived neurotrophic factor (BDNF), changes in hippocampal neurogenesis, HPA axis dysregulation, and circadian rhythm disruption [67, 68]. Notably, these factors do not exist in isolation and frequently influence each other. More specifically, the effects of chronic unpredictable mild stress on the serotonin neural pathway are not new in the literature. Depression-related alterations have been observed in rodents treated with CUMS, such as increased death of 5-HT neurons in the dorsal raphe and the consequent disrupted enervation to the mPFC [69]; impaired frequency/amplitude of excitatory postsynaptic currents induced by serotonin [70]; and decreased 5-HT concentration in the brain, cerebellum and plasma [71]. In this study, CUMS not only reduced BDNF expression but also resulted in significantly downregulated phosphorylation of Akt, PI3K and mTOR in the hippocampus. These manifestations were reversed with treatment. The PI3K/Akt signalling pathway is the primary downstream signalling pathway in BDNF/TrkB signalling, regulating neuronal cell growth and survival in the hippocampus and mediating stress-induced depression and antidepressant effects [72]. Mammalian target of rapamycin (mTOR) is a downstream signalling molecule of the PI3K/Akt pathway that regulates protein translation and synthesis. Depression is caused by synaptic protein defects induced by abnormal mTOR signalling [73]. Recent studies have identified mTOR signalling as one of the targets involved in the rapid antidepressant response. Animal studies detected reduced phosphorylation of mTOR and Akt in the hippocampus of CUMS-exposed mice [74]. Previous studies demonstrated that ketamine increases synaptic protein synthesis by activating the mTOR pathway, increases synaptic function, promotes synapse occurrence and produces rapid antidepressant effects [75]. Similarly, the classical antidepressant fluoxetine modulates the mTOR signalling pathway in the hippocampus of mice exposed to chronic CUMS [76]. These results suggest that the PI3K/Akt/mTOR signalling pathway plays an irreplaceable role in treating depression. We found that CUMS induced depression-like symptoms and reduced PI3K/Akt/mTOR phosphorylation in the hippocampus, which were ameliorated by long-term treatment. Antidepressant SSRIs, such as FLX, which mainly target the central 5-HT transporter, are often prescribed to increase central 5-HT levels in depressed patients.

However, few studies have investigated how the brain is specifically regulated by the gut microbiota. Monoamine oxidase is responsible for converting 5-HT to 5-HIAA [77]. Conversely, Parabacteroides has been reported to produce the tryptophan catabolite indoleacetic acid [72]. Therefore, our findings demonstrate that treatment increased the abundance of bacteria related to 5-HT metabolism, leading to the alteration of neurotransmitter signalling in mice. There are several potential pathways through which the gut microbiota can influence brain function. Microorganisms can influence CNS processes bidirectionally via the vagus nerve, modulating the immune system [73, 78] and tryptophan metabolism [74], along with their ability to synthesize a variety of neurotransmitters and produce metabolites [75]. In our study, treatment repaired gut barrier function, which is associated with gut microbiota dysbiosis and increased 5-HT levels. The potential mechanisms by which probiotics regulate the central nervous system have been explored using different strategies, and it is now clear that probiotics can affect the central nervous system through the “microbiota–gut–brain axis” [79, 80]. Previous studies have shown that probiotics, such as strains of *Bifidobacterium* and *Lactobacillus*, attenuate depressive and anxiety-like behaviours in mice by reshaping the gut microbiota. Interestingly, consistent with a previous study [81], our findings highlighted the antidepressant potential of *Lactococcus lactis* E001-B-8 in mice in response to CUMS. The current results also showed that both *Lactococcus* administration and fluoxetine treatment reversed Tph2 gene expression and 5-HTP levels in the colon. Therefore, our findings indicate that both *Lactococcus lactis* E001-B-8 and fluoxetine may improve colonic 5-HTP synthesis in mice with induced chronic stress, while the underlying mechanisms need further investigation. Growing clinical evidence has revealed that an altered gut microbiome composition is closely associated with neuropsychiatric disorders, including depression. Similarly, alterations in gut microbiome composition in mice exposed to CUMS have been observed. In the current study, CUMS induced evident changes in gut microbiome composition, as reflected by a decreased microbial diversity. Therefore, the gut microbiota can be one of the potential targets for psychobiotics. Consistently, the current study showed that *Lactococcus lactis* E001-B-8 administration reversed the reduced gut microbial diversity and the altered abundances of key taxa in mice with induced chronic stress. In addition, probiotics could also influence the levels of BDNF, gamma-aminobutyric acid, 5-HT, and dopamine in the brain. However, despite these improvements in psychobiotic therapy for depression, clinical applications of probiotics in depression treatment in humans are still limited.

## Conclusions

In conclusion, the stress of males and females was more obvious in CUMS and induced cognitive dysfunction and exacerbated the disorder of the intestinal flora. Supplementation with *Lactococcus lactis* E001-B-8 promotes 5-HT synthesis and can alleviate clinical symptoms. Therefore, *Lactococcus lactis* E001-B-8 may improve CUMS-induced cognitive dysfunction in mice by regulating intestinal flora and increasing 5-HT and provide vital information for developing novel therapeutic treatments.

## Supplementary Information

**Additional file 1: Fig. S1.** CUMS differently affects on the hippocampus between male and female mice. (a–c) Western blotting analysis and quantification data of BDNF, and TrkB in the hippocampus of mice. (d–i) The mRNA expression of BDNF, TrkB, CORT, NE, DA and iNOS in the hippocampus of mice. Each value represents the mean ± SEM. *p < 0.05, **p < 0.01 and ***p < 0.001 means difference of female vs. male mice at the same point.

**Additional file 2: Fig. S2.** Differential gene screening and gene enrichment analysis in data set GSE151807 and KEGG Enrichment Analysis of DEGs. (a) Volcano plot of differentially expressed genes. (b) Hierachical clustering of the differentially expressed genes. (c) Neuroactive ligand–receptor interactions. (d) Ubiquitin-mediated proteolysis. (e) Huntington's disease. (f) Glutathione metabolism. (g) Peroxidase. (h) Long-term depression. (i) Enrichment analysis of differential gene KEGG signaling pathway.

## Data Availability

The data sets used and/or analyzed during the current study are available from the corresponding author on reasonable request.
