# Supplementary figures and images for: 5-HT attenuates chronic stress-induced cognitive impairment in mice through intestinal flora disruption
Source: J Neuroinflammation. 2023 Feb 3;20:23. doi: 10.1186/s12974-023-02693-1 (PMC9896737; doi:10.1186/s12974-023-02693-1)

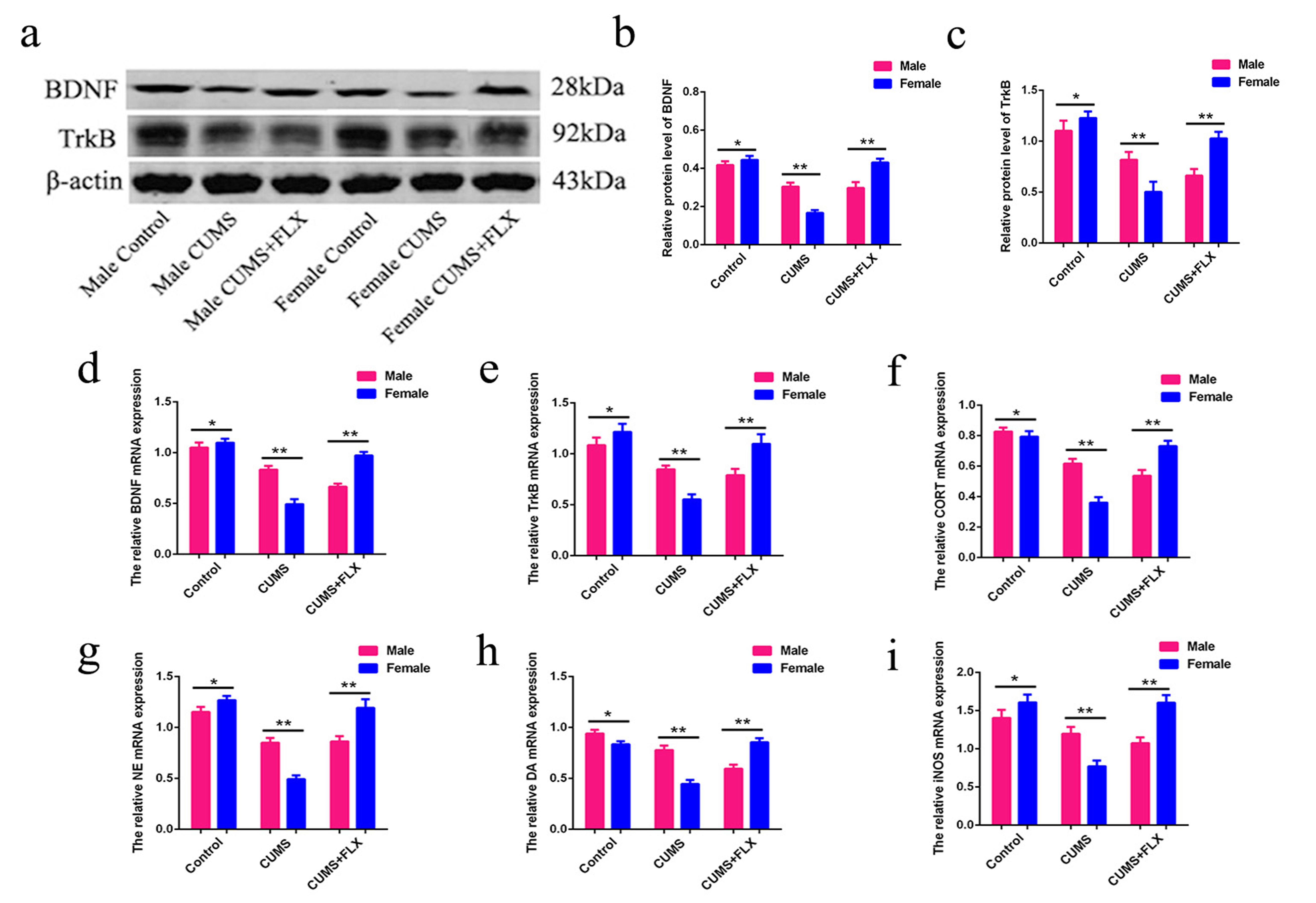

Supplement: Supplementary file 1 — Additional file 1: Fig. S1. CUMS differently affects on the hippocampus between male and female mice. (a–c) Western blotting analysis and quantification data of BDNF, and TrkB in the hippocampus of mice. (d–i) The mRNA expression of BDNF, TrkB, CORT, NE, DA and iNOS in the hippocampus of mice. Each value represents the mean ± SEM. *p < 0.05, **p < 0.01 and ***p < 0.001 means difference of female vs. male mice at the same point. [file 12974_2023_2693_MOESM1_ESM.jpg]

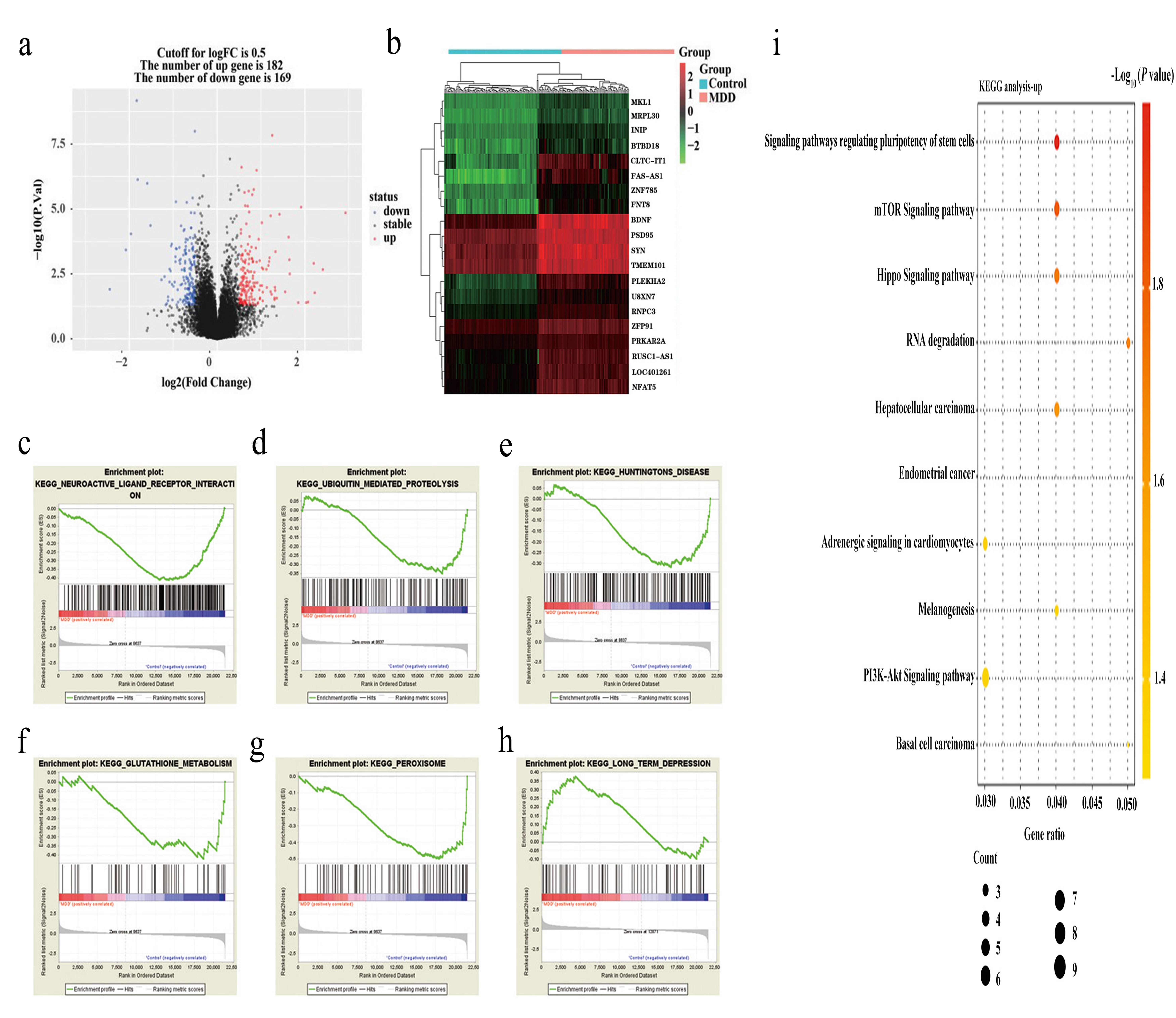

Supplement: Supplementary file 2 — Additional file 2: Fig. S2. Differential gene screening and gene enrichment analysis in data set GSE151807 and KEGG Enrichment Analysis of DEGs. (a) Volcano plot of differentially expressed genes. (b) Hierachical clustering of the differentially expressed genes. (c) Neuroactive ligand–receptor interactions. (d) Ubiquitin-mediated proteolysis. (e) Huntington's disease. (f) Glutathione metabolism. (g) Peroxidase. (h) Long-term depression. (i) Enrichment analysis of differential gene KEGG signaling pathway. [file 12974_2023_2693_MOESM2_ESM.jpg]
